# High mobility group protein B1 is a predictor of poor survival in ovarian cancer

**DOI:** 10.18632/oncotarget.20538

**Published:** 2017-08-24

**Authors:** Lee R. Machado, Paul M. Moseley, Robert Moss, Suha Deen, Christopher Nolan, Ian Spendlove, Judith M. Ramage, Stephen YT. Chan, Lindy G. Durrant

**Affiliations:** ^1^ Faculty of Health and Society, University of Northampton, Boughton Green Road, Northampton, NN2 7AL, United Kingdom; ^2^ Academic Department of Clinical Oncology, Division of Cancer and Stem cells, City Hospital Campus, University of Nottingham, Nottingham NG5 1PB, UK; ^3^ Department of Histopathology, Nottingham University Hospitals NHS Trust, Queen's Medical Centre Campus Division of Clinical Pathology Division of Clinical Oncology, School of Molecular Medical Sciences, University of Nottingham, City Hospital Campus, Nottingham NG5 1PB, UK; ^4^ Clinical Oncology Department, Nottingham University Hospitals, Nottingham NG5 1PB, UK; ^5^ Department of Genetics, University of Leicester, Leicester, LE1 7RH, UK; ^6^ Faculty of Science, Technology, Engineering & Mathematics, The Open University, Milton Keynes, MK7 6AA, UK

**Keywords:** HMGB1, autophagy, ovarian cancer, prognostic, DAMP

## Abstract

High-mobility group protein B1 (HMGB1) has been implicated in numerous tumour types where expression regulates tumour cell growth and survival. We hypothesised that high HMGB1 expression in ovarian tumours would predict poor patient survival.

Using tissue microarrays of primary ovarian cancers combined with a comprehensive database of clinicopathological variables, the expression of HMGB1 was assessed by immunohistochemistry in two independent cohorts (n=194 and n=360) using a monoclonal antibody specific for HMGB1.

Kaplan-Meier analysis showed an association of HMGB1 expression with progression free survival in the primary cohort (p=0.023). In the validation cohort, expression was associated with overall survival (p=0.002). Low expression of HMGB1 was protective and in a multivariate model HMGB1 expression was shown to be an independent predictor of poor survival in ovarian cancer (p=0.006).

The role of HMGB1 in cancer is complex. As high levels of HMGB1 expression are likely to render ovarian cancer cells resistant to chemotherapy, therapies targeting the HMGB1 axis may be appropriate in the treatment of ovarian cancer patients.

## INTRODUCTION

Ovarian cancer is the 5^th^ most common cancer and the 4^th^ most common cause of female cancer deaths in the UK with a European age standardised incidence of approximately 17 cases/100,000 women. Whilst survival from ovarian cancer has almost doubled over the last 30 years, the 5 year survival rate is still relatively poor at less than 50%. The incidence and mortality rate in women over the age of 65 is notably higher (survival rate less than 30%) with patients typically presenting with Stage III/IV metastatic disease [1]. However, if detected at the earliest stages of development, 90% of patients will survive. Therefore, it is vital that novel independent prognostic markers are identified in order to improve our understanding of ovarian cancer biology and the management of these patients.

High-mobility group protein B1 (HMGB1) has recently been implicated in a number of human cancers including colon, gastric, lung, and liver (reviewed extensively in [2]. The role of HMGB1 (a chromatin binding protein) in processes relevant to cancer cell survival include autophagy [3, 4], genome stability [5], angiogenesis [6] and invasion and metastasis [7].

HMGB1 contains a long highly acidic C-terminal tail and two HMG box (A box and B box) domains which mediate DNA binding in a non-sequence specific manner [8]. It is the most ubiquitously expressed of the HMG family members and is able to translocate between the nucleus and cytoplasm [9]. HMGB1 has a complex range of functions depending in part on its subcellular and extracellular localisation, redox state, and interaction with cell surface receptors and transcription factors. Within the nucleus HMGB1 binds chromatin and is involved in DNA repair, replication, recombination, transcription and genomic instability. HMGB1 expression can regulate the mitochondrial bioenergetics of cancer cells by enhancing complex I activity, ATP production and subsequent proliferation and migration of tumour cells [10].

Of recent interest is the role of HMGB1 as a positive regulator of autophagy in cancer [11]. Autophagy is the process of maintaining cellular homeostasis under conditions of stress (i.e. hypoxia and increased genome instability) with the removal of unwanted/damaged cytosolic organelle contents into double membrane structures called autophagosomes. In cancer cells, stress induced autophagy acts in a cyto-protective manner preventing a shift to apoptotic cell death. During cellular stress HMGB1 translocates to the cytosol and binds beclin 1 which subsequently induces the formation of autophagosomes [12]. p53 is a known negative regulator of autophagy and is frequently mutated in ovarian cancer (with near universal mutation in the high grade serous subtype). Deficiency in p53 causes increased expression of cytosolic HMGB1 and enhanced autophagic flux [13].

The prognostic value of HMGB1 in ovarian cancer remains unclear. There is an important need to understand the context-dependent role of HMGB1 as either an anti- or pro-tumorigenic protein in ovarian cancer. To achieve this, the expression and prognostic value of HMGB1 was examined using ovarian cancer tissue microarrays from two independent cohorts. Both cohorts were analysed to determine the effect on survival and the utility of HMGB1 as an independent prognostic marker.

## RESULTS

### HMGB1 protein expression is associated with progression free and overall survival in ovarian cancer cohorts

**Figure 1:**
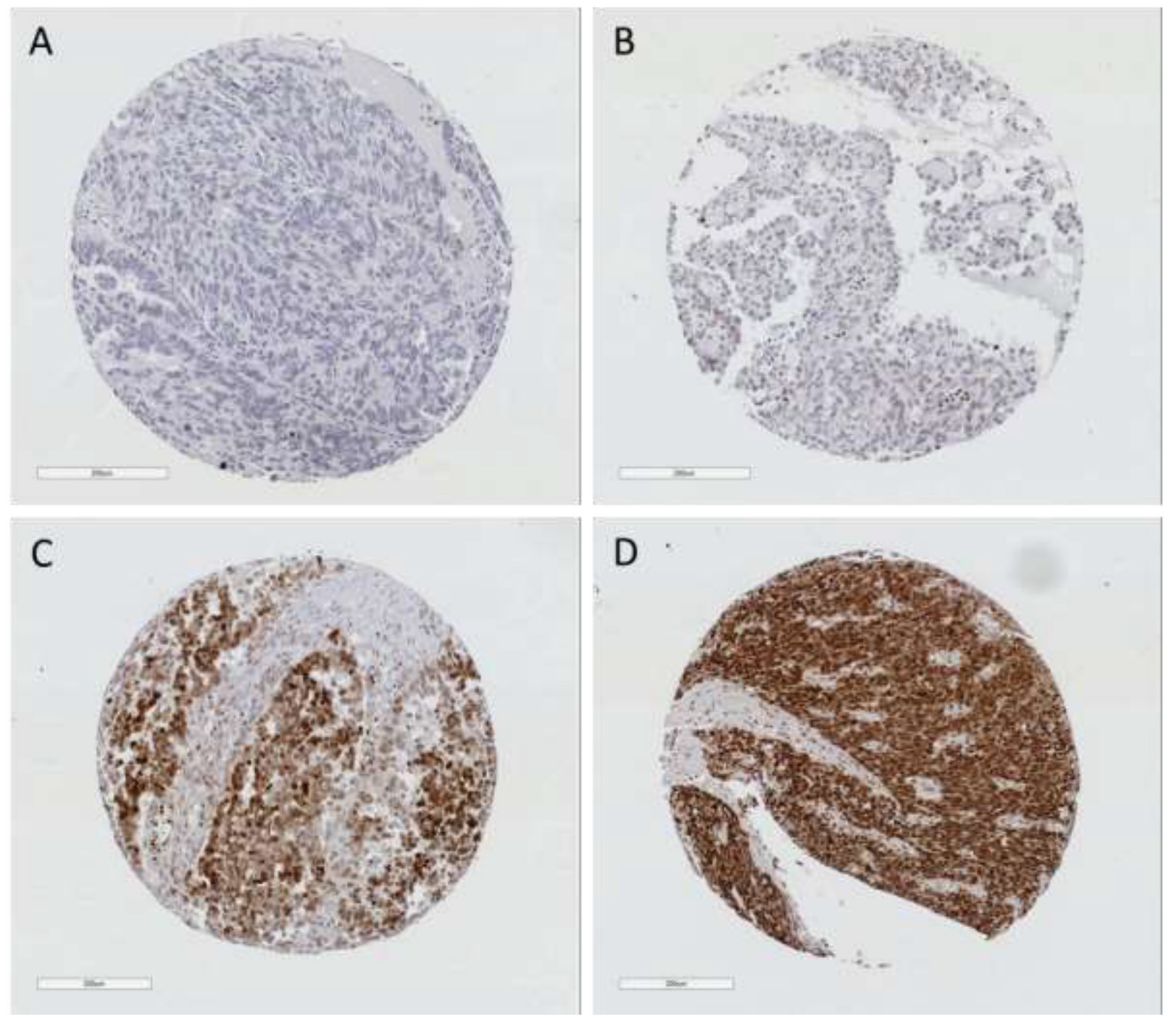
Representative photomicrographs of ovarian TMA cores immunohistochemically stained for HMGB1 from Nottingham cohort (cut point >42) The level of expression ranged from **(A)** Negative (H-score 0), **(B)** Weak (H-score 14), **(C)** Intermediate (H-score 167) and **(D)** Strong expression (H-score 213).

**Figure 2:**
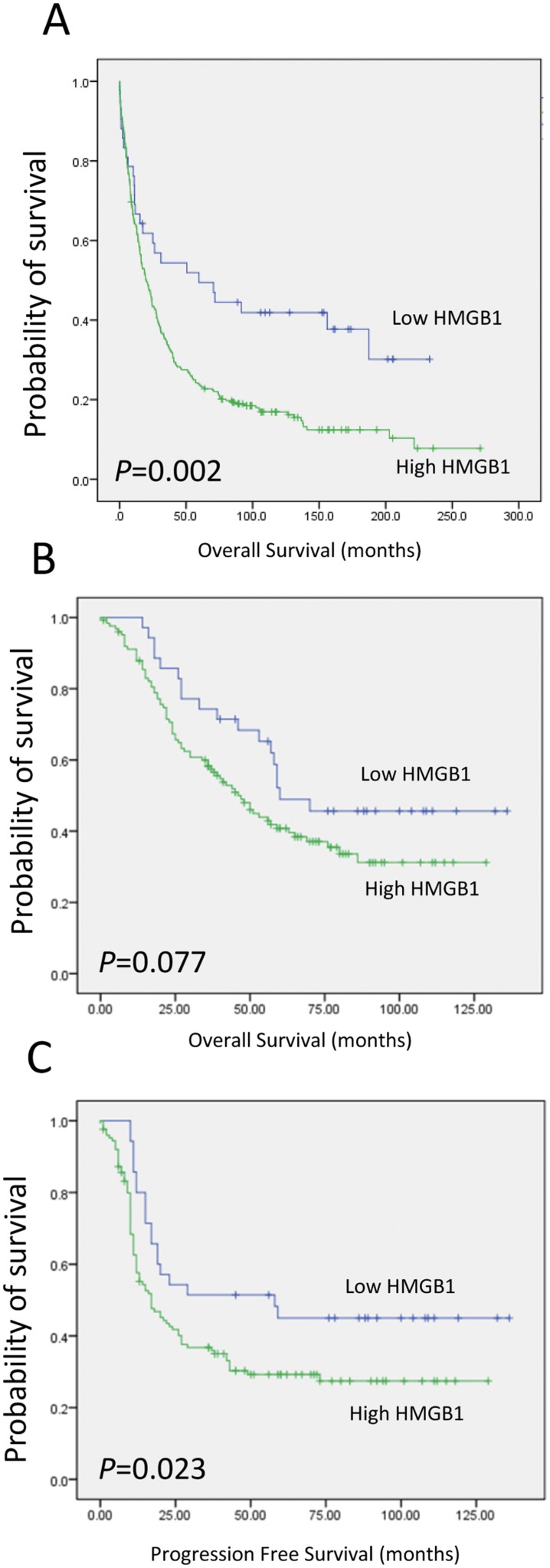
Kaplan Meier curves showing overall survival in **(A)** the Derby cohort and **(B)** overall survival and **(C)** progression free survival in ovarian cancer patients in the Nottingham cohort.

To determine whether the HMGB1 expression was associated with patient survival, we stained a primary (Nottingham) cohort comprised of 194 ovarian cancer cases using immunohistochemistry with a rabbit monoclonal antibody specific for endogenous HMGB1. The monoclonal antibody detected endogenous nuclear and cytoplasmic staining for HMGB1 (Figure 1). Of the 162 evaluable ovarian tumours stained, 35/162 (22%) had weak/absent staining and 127/162 (78%) had positive staining (cut point H-score >42 represented positive expression). Kaplan-Meier survival analysis demonstrated that there was a statistically significant difference between patients that had high or low/absent expressing tumours and progression free survival (log rank test, p=0.023). There was also a trend with overall survival but it failed to reach significance in this primary cohort (p=0.077) (Figure 2).

This finding was expanded on in a validation cohort of 360 ovarian cancer cases with extensive clinical data. Of the 321 evaluable ovarian tumours stained with the HMGB1 specific monoclonal antibody, 43/321 (13%) had weak/absent staining and 278/321 (87%) had positive staining having dichotomised cases into high or low/absent expressing groups. Kaplan-Meier survival analysis demonstrated an association with HMGB1 levels and overall survival for the validation cohort (log-rank test, p=0.002) (Figure 2). Weak/absent expression of HMGB1 expression gave an almost 2 fold increase in survival time from 56 months to 104 months (Table 1). HMGB1 expressing tumours resulted in a poor overall survival outcome.

**Table 1:** Mean survival time in relation to HMGB1 expression

Expression	Mean survival time (months) in relation to HMGB-1 expression			
	Estimate (months)	95% confidence interval		
		Lower bound	Upper bound	*P*-value
Low	104.2	73.2	135.1	
High	55.7	45.4	65.9	0.002
Overall	63.7	53.2	74.1	

### HMGB1 expression is associated with tumour stage, histology, and the administration of adjuvant chemotherapy

Univariate analysis was performed on the validation cohort to determine whether HMGB1 expression was associated with standard clinicopathological variables (Table 2). Pearson's χ^2^ or Fisher's exact tests demonstrated that HMGB1 expression was associated with tumour stage (p=0.050), histological type (p<0.001) and the addition of adjuvant chemotherapy (p=0.002).

**Table 2:** Univariate analysis of HMGB1 expression in association with standard clinicopathological variables and autophagy regulators using the χ^2^-or Fisher's Exact test

Variable	*χ*^2^-test (*P*-value)
	HMGB1
SEER age	0.186^*^
Tumour FIGO stage	0.050^*^
Tumour grade	0.646^*^
Macroscopic residual disease	0.085
Adjuvant therapy	0.005^*^
Histological type	**<0.001**

Abbreviation: FIGO=International Federation of Gynecology and Obstetrics

^*^Fishers exact test *P*-values <0.05 are accepted to be significant.

### HMGB1 represents an independent prognostic marker in ovarian cancer

To determine whether HMGB1 represented an independent prognostic marker, Cox regression was performed (Table 3). HMGB1 was still associated with survival when stage, histology and adjuvant therapy were included as potentially confounding factors in a multivariate Cox regression (with individual Kaplan-Meier statistics of p<0.001, p<0.001 and p<0.033 respectively). In this multivariate model the histological tumour type (p=0.467) was no longer significant. However, HMGB1 remained an independent prognostic factor (p=0.006, HR=1.921). In addition FIGO stage (p<0.001) and response to chemotherapy (p<0.001) also remained independent predictors of patient survival suggesting that HMGB1 may be a useful independent prognostic marker in ovarian cancer.

**Table 3:** Multivariate analysis for overall cancer specific survival in 309 consecutive patients with epithelial ovarian cancer

	Exp(*B*)	95% CI for Exp (*B*)		*P*-value
		Lower	Upper	
***FIGO stage***				
Stage 1	1			<.001
Stage 2	3.350	1.918	5.852	
Stage 3	7.886	4.896	12.704	
Stage 4	10.021	5.810	17.284	
***Histological type***				
				0.467
Borderline	1			
Clear cell OVCA	1.130	0.479	2.667	
Mucinous OVCA	1.695	0.767	3.744	
Endometrioid OVCA	1.339	0.606	2.962	
Serous OVCA	1.594	0.796	3.195	
Undifferentiated OVCA	1.598	0.761	3.354	
Other OVCA	3.180	0.960	10.533	
***Adjuvant therapy***				
No	1			<.001
Yes	0.361	0.240	0.541	
***HMGB1***				
Low	1			0.006
High	1.921	1.205	3.064	

Abbreviations: CI=confidence interval; FIGO=International Federation of Gynecology and Obstetrics.

The analysis is based on a Cox multivariate regression model. *P*-values <0.05 are accepted to be significant.

## DISCUSSION

To our knowledge this work represents the first large scale tissue microarray analysis of HMGB1 protein expression in multiple subtypes of ovarian cancer with replication in an independent second cohort. Our results indicate that high expression of HMGB1 is deleterious in ovarian cancer reducing the overall mean survival time (from 104 months in low/absent expressing tumours to 56 months in high expressing tumours). The role of HMGB1 in cancer is complex and is likely to be tumour cell specific as well as contingent on the redox state of HMGB1, its subcellular localisation and the expression of corresponding ligands/binding partners (reviewed in Kang *et al*, 2013). In our study, there was a trend towards HMGB1 expression being associated with overall survival in the primary cohort. However, HMGB1 expression was associated with progression free survival in the primary cohort. Indeed, our data support the results of a recent small scale study of ovarian cancer patients (n=74 patients) which confirmed that high HMGB1 tissue expression correlated with progression free survival [14]. We extend these observations by demonstrating that HMGB1 was associated with overall survival in our validation cohort.

The mechanism by which HMGB1 expression is deleterious for ovarian cancer patients is currently unclear. HMGB1 may play a role in the bioenergetic output of the tumour. Recent data in pancreatic cell lines suggests HMGB1 (in combination with one of its receptors RAGE) may regulate mitochondrial complex I activity, and subsequent ATP production, with a resulting increase in cellular proliferation and migration [10]. However, to date no studies have explored how HMGB1 may regulate the bioenergetics of ovarian cancer cells and this will be a fruitful area for further investigation.

HMGB1 may play an important role in shifting the balance from apoptosis to autophagy. HMGB1 and wild type p53 regulate the expression of each other [15, 16]. Furthermore HMGB1 has been reported to form a complex with wild type p53 regulating the balance between apoptosis and autophagy [13]. In p53 deficient HCT116 cells cytosolic expression of HMGB1 is increased inducing autophagy. Therefore, HMGB1 promotes autophagy in the setting of diminished wild type p53. Although beyond the scope of this study, it will be important to determine whether expression of HMGB1 in ovarian cancer is associated with other markers of autophagy including LC3B and Beclin-1.

Our work has some limitations as we were unable to determine the relative proportions of HMGB1 in different subcellular compartments. Furthermore, our antibody reagent does not discriminate (to our knowledge) the different redox states of HMGB1 or acetylation status of HMGB1, all of which are known to influence HMGB1 function [17, 18].

Our finding that HMGB1 expression levels in the validation cohort were associated with the administration of adjuvant chemotherapy and both remain independent prognostic markers after multivariate analysis is an interesting finding and has implications for therapy. Whilst our data indicates an association between HMGB1 and administration of therapy, individual Kaplein-Meier statistics for the addition of chemotherapy indicate that in this cohort patients who received no chemotherapy had a better survival outcome than those that did (p=0.033 data not shown). The reasons for this are unclear but may reflect the proportion of patients given chemotherapy between 1984 and 1997, with patients with a worse prognosis receiving this treatment. Patient numbers were insufficient for survival analysis grouping patients based on both chemotherapy and HMGB1 co-status. Nevertheless, our data indicates that HMGB1 is associated with poor prognosis in two cohorts spaced 10 years apart with different proportions treated with chemotherapy.

Previous *in vitro* experiments assessing chemosensitivity of ovarian cancer cells lines (to cisplatin) have shown that they are dependent on the BTP/POZ transcription family member NAC1 which induces autophagy [19]. Under stress (i.e. that induced by cytotoxic agents) NAC1 increases expression and cytosolic translocation of HMGB1. More cytosolic HMGB1 is then available to bind beclin-1 (and displace it from Bcl-2) and subsequently induce autophagy. NAC-1 knockdown experiments in ovarian cancer cell lines treated with cisplatin resulted in a reduction of cisplatin/HMGB1 induced autophagy and an increase in cytotoxicity of ovarian cancer cells. Therefore, HMGB1 protects ovarian cancer cells from chemotherapy induced apoptosis by shifting the balance from apoptosis to autophagy [20].

Our work using two independent cohorts of ovarian cancer patients demonstrates that HMGB1 may represent an independent marker of poor prognosis. Supporting a role for HMGB1 as an independent prognostic marker, a study of HMGB1 serum levels in 105 ovarian carcinoma patients demonstrated that increased levels of HMGB1 were found in patients with more advanced stage disease and were at higher levels in cancer patients compared with healthy controls [21]. Therefore, future functional studies should determine the mechanism of HMGB1 function in ovarian cancer. If this can be determined, then strategies designed to modulate the HMGB1 pathway may provide a rational approach in the therapeutic targeting of ovarian cancer.

## MATERIALS AND METHODS

### Patient samples

This is a retrospective validation study with patients comprehensively staged according to the International Federation of Obstetricians and Gynecologists (FIGO) staging system for ovarian cancer. Full pathology review has been performed to current standards. Clinical details of both the Nottingham (primary cohort) (n=194) [22] and Derby (validation cohort) (n=360) [23] cohorts have been previously described (Supplementary Tables 1 and 2). This work was approved by the Derby Royal Hospital Ethics Committee and Nottingham Research Ethics Committee. In the compilation of this manuscript, the Reporting Recommendations for Tumor Marker Prognostic Studies (REMARK) criteria, were followed throughout the study [24].

#### Nottingham University Hospitals cohort (Primary)

The cohort comprises 194 consecutive cases of ovarian epithelial cancer treated at Nottingham University Hospitals from 2000 to 2007. Survival was calculated from the operation date until 1st of October 2012 when any remaining survivors were censored.

#### Derby City Hospital cohort (Validation)

The cohort consisted of 360 consecutive ovarian epithelial cancer cases treated at Derby City Hospitals between 1^st^ January 1984 and 31^st^ December 1997. Survival was calculated from the operation date until 31 November 2005 when any remaining survivors were censored. The database was audited to ensure validity; there were no major discrepancies with over 97% of data available.

### Tissue microarray and immunohistochemistry

Tissue microarrays were constructed as described previously [22, 23, 25]. Antibodies were optimised on full faced sections. Positive and negative (omission of the primary antibody and replacement with IgG-matched serum) controls were included in each run. Immunohistochemical staining was performed using a routine streptavidin–biotin peroxidase method. Tissue-array sections were first deparaffinised with xylene, rehydrated through graded alcohol and immersed in methanol containing 0.3% (v/v) hydrogen peroxide for 20 minutes. Antigen retrieval was achieved by immersing sections in pH 6.0 citrate buffer and heated in an 800W microwave for 10 minutes at high power and 10 minutes at low power. Endogenous avidin/biotin binding was blocked (avidin/biotin blocking kit, Vector Labs, Peterborough, UK), followed by addition of 100 μl 1:5 normal swine serum (NSS) to Phosphate buffered saline (PBS) for 15 minutes to block non-specific binding.

Sections were incubated with a Rabbit anti-HMGB1 mAb (clone D3E5) (New England Biolabs, Hitchin, UK). After washing with PBS, sections were incubated with 100 μl of biotinylated goat anti-mouse/rabbit immunoglobulin (Dako Ltd, Ely, UK) diluted 1:100 in NSS, for 30 minutes, washed in TBS and incubated with 100 μl of pre-formed streptavidin–biotin/horseradish peroxidase complex (Dako) for 60 minutes. Visualisation was achieved using 3,3′-diaminobenzidine tetra hydrochloride (DAB, Dako), and then lightly counterstained with haematoxylin (Dako), dehydrated in alcohol, cleared in xylene, and mounted with distyrene, plasticiser and xylene (DPX–BDH, Poole, UK).

### Evaluation of staining

Tumour microarray cores were imaged using a NanoZoomer (Hamamatsu, Bridgewater, NJ, USA). Image analysis was semi-automated using Aperio ImageScope v11.2.0.780 software. The positive pixel count v9.1 algorithm settings were modified to provide accurate calling of expression levels (Iwp(high) at 140, Iwp(Low)=Ip (High) at 103 and Ip (low)=Isp(High) at 75). The accuracy of these settings to correctly call expression levels was confirmed by two experienced observers in the analysis of TMAs. Tumours were assigned a modified Histo-score (H-score) and assessed for high, low or negative HMGB1 expression. Observers were blind to clinical and pathological parameters. X-TILE software was employed to determine cut point selection values to segregate tumours into high and low/absent expressing groups [26].

### Statistical analysis

Statistical analysis was performed using SPSS20 statistical software (SPSS Inc., Chicago, IL, USA). Pearson's χ^2^-tests and Fisher's exact tests were used to determine the significance of associations between categorical variables. Survival rates were calculated using the Kaplan–Meier method; differences between survival curves were tested using the log-rank test. The Cox proportional-hazards model was used for multivariate analysis in order to calculate the Hazard ratios and independent significance of individual factors. In all cases two-sided P-values of <0.05 were considered as statistically significant.

## SUPPLEMENTARY MATERIALS FIGURES AND TABLES



## Abbreviations

(HMGB1): High-mobility group protein B1
(TLR): Toll-like receptor
(STAT1): Signal transducer and activator of transcription 1
(FIGO): Federation of Obstetricians and Gynecologists
(REMARK): Reporting Recommendations for Tumor Marker Prognostic Studies
(Bcl-2): B-cell lymphoma-2
(Nac-1): nuclear accumbens-1
(TMA): Tissue microarray
(mAb): Monoclonal antibody
(DAB): 3,3′-diaminobenzidine tetra hydrochloride
