# Supplementary material for: High mobility group protein B1 is a predictor of poor survival in ovarian cancer
Source: Oncotarget. 2017 Aug 24;8(60):101215–23. doi: 10.18632/oncotarget.20538 (PMC5731868; doi:10.18632/oncotarget.20538)
Supplement: Supplementary file 1 [file oncotarget-08-101215-s001.pdf]

High mobility group protein B1 is a predictor of poor survival in ovarian cancer

SUPPLEMENTARY MATERIALS

Supplementary Table 1: Clinicopathological variables for the Nottingham patient cohort (n=194) and cores stained for HMGB1

Variable	Categories	Frequency of total cohort (%), n= 194	Frequency of the HMGB1-stained cohort (%), *n*= 162
Cohort age characteristics	<30 years at diagnosis	1 (1)	1 (1)
	30–60 years at diagnosis	90 (46)	76 (47)
	>60 years at diagnosis	101 (52)	85 (52)
	Unknown	2 (1)	0 (0)
Optimal Debulking at Surgery	Yes	124 (64)	109 (67)
	No	67 (35)	52 (32)
	Unknown	3 (2)	1 (1)
Macroscopic residual disease	Absent	60 (31)	54 (33)
	Present	133 (69)	108 (67)
	Unknown	1 (1)	0 (0)
FIGO stage	I	59 (30)	50 (31)
	II	27 (14)	24 (15)
	III	85 (44)	70 (43)
	IV	21 (11)	18 (11)
	Unknown	2 (1)	0 (0)
Histological type	Serous carcinoma	111 (57)	94 (58)
	Mucinous cystoadenocarcinoma	14 (7)	11 (7)
	Endometrioid	38 (20)	33 (20)
	Clear cell	24 (12)	19 (12)
	Undifferentiated	0 (0)	0 (0)
	Others	3 (2)	3 (2)
	Unknown	4 (2)	2 (1)
Chemotherapy	Interval debulking	56 (29)	47 (29)
	Adjuvant	123 (63)	103 (64)
	Not indicated	14 (7)	12 (7)
	Unknown	1 (1)	0 (0)

Supplementary Table 2: Clinicopathological variables for the Derby patient cohort (n=360) and cores stained for HMGB1

Variable	Categories	Frequency of total cohort (%), n=360	Frequency of the HMGB1-stained cohort (%), *n*=321
SEER age characteristics	<30 years at diagnosis	2 (<1)	1 (<1)
	30–60 years at diagnosis	143 (40)	127 (40)
	>60 years at diagnosis	212 (59)	190 (59)
	Unknown	3 (<1)	3 (1)
Macroscopic residual disease	Absent	143 (40)	126 (39)
	Present	201 (56)	180 (56)
	Unknown	16 (4)	15 (5)
FIGO stage	I	95 (26)	88 (27)
	II	38 (11)	34 (11)
	III	175 (49)	155 (48)
	IV	40 (11)	33 (10)
	Unknown	12 (3)	11 (3)
Histological type	Serous carcinoma	178 (49)	159 (50)
	Mucinous cystoadenocarcinoma	35 (10)	31 (10)
	Endometrioid	42 (12)	39 (12)
	Clear cell	25 (7)	25 (8)
	Undifferentiated	54 (15)	47 (15)
	Others	26 (7)	20 (6)
Adjuvant therapy	No	101 (28)	92 (29)
	Yes	249 (69)	220 (69)
	Unknown	10 (3)	9 (3)

Supplementary Table 3: Univariate analysis of HMGB-1 expression (Derby cohort) in association with standard clinicopathological variables using the χ2-test or Fisher's Exact test

Variable	Low	High	P value
SEER Age (n=318)			
<30 years	1 (0.3%)	0 (0.0%)	
30-59 year	16 (5%)	111 (34.9%)	0.186*
>60 years	25 (7.9%)	165 (51.9%)	
FIGO Stage (n=310)			
I	18 (5.8%)	70 (22.6 %)	
II	5 (1.6%)	29 (9.4%)	**0.050***
III	13 (4.2%)	142 (45.8)	
IV	3 (1.0%)	30 (9.7%)	
Macroscopic residual disease (n=321)			
Unknown	4 (1.2%)	11 (3.4%)	
<2cm	10 3.1%)	73 (22.7%)	0.123
>2cm	8 (2.5%)	89 (27.7%)	
None	21 (6.5%)	105 (32.7%)	
Macroscopic residual disease (n=306)			
Absent	21 (6.9%)	105 (34.3%)	0.085
Present	18 (5.9%)	162 (52.9%)	
Histological type (lethalness) (n=319)			
Borderline	1 (0.3%)	12 (3.8%)	
Clear cell	11 (3.4%)	14 (4.4%)	
Mucinous	6 (1.9%)	25 (7.8%)	
Endometroid	5 (1.6%)	34 (10.7%)	**<0.001**
Serous	10 (3.1%)	149 (46.7%)	
Undifferentiated	8 (2.5%)	39 12.2%)	
other OVCA	1 (0.3%)	4 (1.3%)	
Type of Adjuvant therapy (n=321)			
Unknown	3 (0.9%)	6 (1.9%)	
Carboplatin/taxol	1 (0.3%)	5 (1.6%)	
Carboplatin	7 (2.2%)	71 (22.1%)	**0.023***
Non-platinum	6 (1.9%)	62 (19.3%)	
Nothing	18 (5.6%)	69 (21.5%)	
Platinum	6 (1.9%)	62 (19.3%)	
Radiotherapy	2 (0.6%)	3 (0.9%)	
Type of Adjuvant therapy (n=312)			
No chemotherapy	20 (6.4%)	72 (23.1%)	**0.002**
Platinum/non platinum	20 (6.4%)	200 (64.1%)	
Status (n=316)			
Alive	16 (5.1%)	41 (13.0%)	**<0.001**
Dead	26 (8.2%)	233 (73.7%)	
